# Bioengineered Pancreas–Liver Crosstalk in a Microfluidic Coculture Chip Identifies Human Metabolic Response Signatures in Prediabetic Hyperglycemia

**DOI:** 10.1002/advs.202203368

**Published:** 2022-10-26

**Authors:** Reza Zandi Shafagh, Sonia Youhanna, Jibbe Keulen, Joanne X. Shen, Nayere Taebnia, Lena C. Preiss, Kathrin Klein, Florian A. Büttner, Mikael Bergqvist, Wouter van der Wijngaart, Volker M. Lauschke

**Affiliations:** ^1^ Department of Physiology and Pharmacology Karolinska Institutet Stockholm 17711 Sweden; ^2^ Division of Micro‐ and Nanosystems KTH Royal Institute of Technology Stockholm 10044 Sweden; ^3^ Dr Margarete Fischer‐Bosch Institute of Clinical Pharmacology 70376 Stuttgart Germany; ^4^ University of Tuebingen 72074 Tuebingen Germany; ^5^ Department of Drug Metabolism and Pharmacokinetics (DMPK) The Healthcare Business of Merck KGaA 64293 Darmstadt Germany

**Keywords:** glycemic control, microfluidic cell culture, microphysiological model, organ‐on‐a‐chip, tissue interaction

## Abstract

Aberrant glucose homeostasis is the most common metabolic disturbance affecting one in ten adults worldwide. Prediabetic hyperglycemia due to dysfunctional interactions between different human tissues, including pancreas and liver, constitutes the largest risk factor for the development of type 2 diabetes. However, this early stage of metabolic disease has received relatively little attention. Microphysiological tissue models that emulate tissue crosstalk offer emerging opportunities to study metabolic interactions. Here, a novel modular multitissue organ‐on‐a‐chip device is presented that allows for integrated and reciprocal communication between different 3D primary human tissue cultures. Precisely controlled heterologous perfusion of each tissue chamber is achieved through a microfluidic single “synthetic heart” pneumatic actuation unit connected to multiple tissue chambers via specific configuration of microchannel resistances. On‐chip coculture experiments of organotypic primary human liver spheroids and intact primary human islets demonstrate insulin secretion and hepatic insulin response dynamics at physiological timescales upon glucose challenge. Integration of transcriptomic analyses with promoter motif activity data of 503 transcription factors reveals tissue‐specific interacting molecular networks that underlie *β*‐cell stress in prediabetic hyperglycemia. Interestingly, liver and islet cultures show surprising counter‐regulation of transcriptional programs, emphasizing the power of microphysiological coculture to elucidate the systems biology of metabolic crosstalk.

## Graphical abstract

Hyperglycemia caused by dysfunctional interactions between pancreas and liver is the largest risk factor for type 2 diabetes. By coculture of primary human liver spheroids and intact primary human pancreatic islets in a microfluidic device with heterologous perfusion, the authors recapitulate functional human tissue crosstalk ex vivo and reveal the molecular networks that underlie prediabetic glycemic stress.

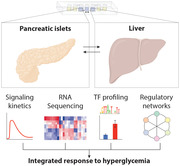

## 1 Introduction

Diabetes mellitus (DM) is the most prevalent metabolic disorder affecting 463 million people or around 1 in 11 adults globally.^[^ ^1^ ^]^ DM can be subclassified into multiple different types based on clinical parameters and etiology;^[^ ^2^, ^3^, ^4^ ^]^ however, irrespective of the used classification system, type 2 DM (T2DM) is by far the most common, accounting for 90–95% of all DM cases. Although diabetes care has improved, complications are still common, and T2DM remains a leading cause of cardiovascular morbidity, visual loss, amputation, and end‐stage renal disease.^[^ ^5^ ^]^ Diabetes also reduces life expectancy by ≈6 years and accounts for 11% of all deaths in the general US population and for 19% in obese persons, demonstrating the necessity of improved disease management.^[^ ^6^, ^7^ ^]^

T2DM is commonly preceded by prediabetes, a state of abnormal glucose homeostasis in which the perturbations are not yet high enough to meet the criteria for a diabetes diagnosis. Prediabetes is associated with pathological alterations in various tissues, including pancreas, and liver, and is commonly defined as intermediate hyperglycemia following an oral glucose tolerance test.^[^ ^8^, ^9^ ^]^ However, while prediabetes constitutes one of the most predictive risk factors for T2DM with annual conversion rates of 5–20%,^[^ ^10^ ^]^ this early stage of metabolic disturbances has received comparably little attention.

This paucity of studies is at least in part due to a lack of experimental tools that faithfully reflect human diabetes pathobiology. Animal models are most commonly used to study T2DM and its common comorbidities, such as nonalcoholic fatty liver disease (NAFLD). However, rodents feature pronounced species‐differences in metabolic traits, and none of the chemically induced, genetic or dietary animal models recapitulate the full spectrum of diabetes and prediabetes manifestations observed in humans.^[^ ^11^ ^]^ Additionally, circadian rhythm has been shown to be an important modulator of insulin sensitivity and glucose tolerance, and as rodents are nocturnal, the reliability of daytime experiments remains questionable.^[^ ^12^ ^]^ Due to their limitations in translatability and throughput, animal experiments are typically complemented by in vitro studies utilizing human cells. Yet, the physiological relevance of the utilized cell models is often questionable, due to the use of transformed immortalized cell lines without thorough and stringent benchmarking to their primary tissue counterparts. When primary human cells are used, they are often cultured in 2D monolayers on stiff surfaces, which do not reflect the in vivo microenvironment of an intact organ. Consequently, the phenotype of the utilized cells rapidly deteriorates for some organs, such as the liver, within a few hours.^[^ ^13^ ^]^

In recent years, organotypic 3D culture models in which human cells can retain molecularly, functionally, and metabolically relevant phenotypes for up to multiple weeks have received increasing attention.^[^ ^14^ ^]^ For the liver, 3D liver spheroid cultures of primary human hepatocytes (PHH) have demonstrated superior phenotypes and functional stability compared to isogenic 2D monolayers or sandwich culture, or when expression signatures are benchmarked to other hepatic cell models such as induced pluripotent stem cell (iPSC)‐derived hepatocyte‐like cells and HepaRG cells.^[^ ^15^, ^16^, ^17^, ^18^ ^]^ Furthermore, PHH spheroids maintain the metabolic configuration of the intact liver^[^ ^19^ ^]^ and we have previously established culture conditions in which PHH liver spheroids retain insulin sensitivity and physiological patterns of glucose utilization.^[^ ^20^, ^21^ ^]^ Similarly, isolated human pancreatic islets can be cultured ex vivo for extended periods of time. As intact islets show remarkable size heterogeneity, protocols have been developed to generate pseudoislets via dissociation and subsequent reaggregation of defined islet cell numbers.^[^ ^22^ ^]^ While the resulting pseudoislets continue to secrete insulin for multiple days, islet dissociation disrupts *β*‐cell hubs consisting of specialized cells with high connectivity, which control islet cell population dynamics and are required for the coordinated response of islets to glucose.^[^ ^23^, ^24^ ^]^

Microphysiological systems provide appealing possibilities to fluidically connect separate culture chambers containing models of different human tissues to emulate and study tissue crosstalk. While an increasing number of microfluidic multitissue devices have been presented in recent years, only few have focused on constellations to emulate key features of T2DM, such as glucose response and glycemic control. Previous models used microfluidic cocultures of primary rat islets and 2D cultures of primary rat hepatocytes,^[^ ^25^ ^]^ reaggregated human pseudoislets, and HepaRG spheroids^[^ ^26^ ^]^ or iPSC‐derived liver and islet models^[^ ^27^ ^]^ to emulate pancreas–liver crosstalk. In addition, elegant technical solutions have been presented for the microfluidic evaluation of insulin secretion dynamics using monocultures of pancreatic cell lines^[^ ^28^ ^]^ or primary human islets.^[^ ^29^ ^]^ While these systems have provided convincing proof‐of‐concept that interactions between islets and liver can be emulated using microfluidic systems, no systems have been presented that model the interaction between organotypic primary human liver and islet cultures. Furthermore, none of these studies have comprehensively profiled the molecular responses to glucose challenge and the complex multifactorial interaction networks that constitute an integral part of T2DM pathobiology.

Here, we present a novel pneumatically actuated microphysiological device that enables efficient crosstalk between 3D primary human liver cultures and intact human pancreatic islets in a perfused environment. Whereas most microfluidic multi‐tissue models feature sequential perfusion of the interconnected compartments, we present a modular system in which the different tissues are actuated simultaneously followed by controlled integration of tissue‐specific signals, enabling reciprocal crosstalk at physiological time scales. Glucose challenge of the integrated coculture chip resulted in rapid secretion of insulin by the islets, followed by hepatic AKT phosphorylation and repression of gluconeogenesis without extrinsic hormone loading. Comprehensive profiling of tissue‐specific on‐chip response signatures to prediabetic hyperglycemia using RNA‐Seq showed rapid activation of molecular hallmarks of the unfolded protein response (UPR) and initiation of regenerative programs in islets. Furthermore, analyses of binding motif activities of 503 transcription factors implicated activation of CEBPB and ONECUT1, as well as inhibition of the key pancreatic differentiation factors MAFA, PTF1A, and RFX in these *β*‐cell stress signatures. In liver cultures, we identified surprising counter‐regulation in gene signatures between liver and islet cultures in response to a high glucose bolus involving PPM1K, which catalyzes the rate‐limiting step of branched chain amino acid (BCAA) catabolism, and the orphan nuclear receptor NR4A1. Combined, these results show that prediabetic glucose concentrations result in rapid islet exhaustion, metabolic alterations, and the initiation of regenerative programs, demonstrating the added value of microphysiological coculture systems for the identification of tissue‐specific regulatory networks.

## 2 Experimental Section

### 2.1 Device Fabrication

Microfluidic chips were assembled from different parts fabricated from off‐stoichiometric thiol–ene–epoxy (OSTE+), poly(methyl methacrylate) (PMMA) and polydimethylsiloxane (PDMS). OSTE+ parts were produced from OSTEMER‐322 (Mercene labs, Sweden) as previously described.^[^ ^30^, ^31^ ^]^ Briefly, thiol and epoxy monomers containing a UV‐sensitive photoinitiator and a heat‐sensitive initiator were mixed by centrifugation in a 1.09:1.00 ratio at 2000 rpm for 3 min using a speedmixer (Hauschild Engineering, Germany). Before use, all aluminum molds were coated with Teflon AF 1601 (Chemours, USA) diluted with fluorinert FC‐40 (Sigma‐Aldrich, Germany) at the ratio of 1:10, to improve the demolding process. Each mold contained two ports, which allowed injection of the polymer into the closed mold while also compensating for shrinkage during polymerization. During production, OSTE+ was either injected or cast into the mold and the mold was subsequently covered with a flat PMMA lid. To prevent OSTE+ from bonding with the PMMA lid, a nonadhesive liner (ScotchPak 9775 Release Liner, 3M, USA) was used. Subsequently, the mold was exposed to a 70 W UV‐light (TLG Technology) for 100–240 s, depending on the thickness of the mold. After UV‐curing, the intermediately OSTE+ was demolded and thermally cured overnight at 65 °C.

Prototypes were produced using a CO_2_ (Universal Laser Systems) laser to etch features into a 75 × 25 × 0.5 mm PMMA block. After etching, a 75 × 25 × 0.5 mm intermediately OSTE+ layer was produced using an aluminum mold. The layer was clamped to the bottom of the chip and thermally cured overnight at 65 °C to bind the OSTE+ to the PMMA, hence sealing off the chambers and channels.

For functional testing devices were produced using micromilling or reaction injection molding (RIM). For micromilling, 75 × 25 × 5 mm OSTE+ blocks were produced using an aluminum mold. The blocks were baked at 220 °C for 12 min and rinsed with water to remove any remaining liquid residues. Liquid OSTE 322 resin was poured into the mold and irradiated using UV‐light for 240 s to start the first polymerization reaction. The polymerized blocks were then demolded and thermally cured overnight at 65 °C to complete the second polymerization reaction. Alternatively, features were milled into the OSTE+ blocks using a MiniMill GX micromilling machine (Minitech Machinery Corp., USA). A 75 × 25 × 0.5 mm intermediately polymerized OSTE‐322 layer was bonded to the bottom of the chip and thermally cured as described above. For RIM‐based fabrication, a Teflon‐coated aluminum mold was clamped to a PMMA lid with a layer of nonadhesive liner (ScotchPak 9775 Release Liner, 3M, USA) and OSTE‐322 was injected through the inlet channels using a 10 mL syringe (Hencke Sass Wolf). The mold was exposed to UV through the transparent PMMA lid for 180–220 s. Afterward, the OSTE+ chip was demolded and a 75 × 25 × 0.5 mm intermediately polymerized OSTE‐322 layer was clamped to the bottom of the chip and thermally cured at 65 °C overnight.

### 2.2 Modeling and Formulation

Flow rates into and out of the satellite chambers can be calculated based on the hydraulic resistances of the connecting channels. For a channel with rectangular cross‐sectional hydraulic resistance *R*_h_ is given by

(1) $$R_{h}=\alpha \approx \frac{12\mu L}{d_{1}d_{2}^{3}\left(1-0.63d_{2}/d_{1}\right)}:d_{2}<d_{1}$$

with *μ* and *L* as the dynamic viscosity of the fluid and the length of the channel, respectively.^[^ ^32^ ^]^ The dimensions of the rectangular cross‐section of channel *i* is given by *d_i_*. Thus, the hydraulic resistance of the channel connected to the *i*th satellite chamber can be estimated as

(2) $$R_{i}\approx \alpha L_{i}$$

where *L_i_* is the length of the channel and *α* is the same for all the channels due to their equal cross‐section (*w* and *h*) and dynamic viscosity of the fluid flowing through them. By virtue of the oil‐lock system, the air pressure inside the satellite chambers is maintained close to 1 atm. Therefore, the pressure drop (Δ*P* = *P*_central_ − *P*_atm_) along the channels, based on the Hagen–Poiseuille's law, is defined as

(3) $$\Delta P=R_{i}Q_{i}=\alpha L_{i}Q_{i}$$

where *Q_i_* is the volumetric flow rate through the *i*th channel. The same pressure gradient also drives the flow between the central chamber and the network of satellite chambers which results in

(4) $$\Delta P=R_{m}Q_{c}=\frac{Q_{c}}{\sum_{i=1}^{n}R_{i}^{-1}}$$

where *R*_m_ is the equivalent hydraulic resistance of the network (which is the parallel sum of all the channels’ resistances) and *Q*_c_ is the volumetric flow rate into and out of the central chamber. Substituting *R_i_* from Equation (2) results in

(5) $$\Delta P=\frac{\alpha Q_{c}}{\sum_{i=1}^{n}{\left(L_{i}\right)}^{-1}}$$

Equating Equations (3) and (5), the volumetric flow rate was obtained through each (*j*th) channel as

(6) $$Q_{j}=\frac{Q_{c}}{L_{j}\sum_{i=1}^{n}{\left(L_{i}\right)}^{-1}}$$

This enables us to estimate the volumetric flow rate for each of the straight or meandering channels based on the volumetric flow rate of the central chamber and the length of all channels of the network. See the Supporting Information for additional modeling details.

### 2.3 Quantification of Mixing Kinetics

To determine the fluid displacement within each chamber, pneumatic actuation was recorded using a digital USB microscope (Celestron). The microscope recorded a lateral view of the three central chambers and was centred on the interface between the OSTE+ chip and the top PMMA layer. Fluid displacement was measured as the difference in volume between the filled and empty state of each chamber during each cycle. The volume in each chamber was calculated by measuring the distance from the fluid level to the bottom of the chamber.

### 2.4 COMSOL Modeling

We developed a time‐dependent numerical simulation of fluid dynamics and mass transport to calculate the optimal flow rates in the microfluidic device using the commercial software package COMSOL Multiphysics 6.0 (COMSOL, Stockholm, Sweden). The model is based on the finite element method (FEM) and solves transient partial differential equations of linear momentum, mass transport, and diffusion over the entire computational domain. See the Supporting Information for details of the model. Surface‐tracking of the deformable liquid/gas interfaces was carried out based on the moving mesh approach. For model optimization, fluid displacement, and its associated redistribution of solutes between the different compartments were modeled and compared to experimental data. Shear stress on liver spheroids and pancreatic islets were modeled by assuming spherical structures of 200 and 100–300 µm, respectively, for different positions in the respective chambers at flow rates of 50, 100, 500, 1000, 2000, and 4000 µL min^−1^.

### 2.5 Organotypic Cell Culture

Human liver spheroids were formed using primary human hepatocytes (PHH) acquired from BioIVT (Maryland, USA). The supplier received informed consent from each donor or the subject's legally authorized representative. The informed consent forms as well as the relevant protocols were reviewed and approved by the appropriate regulatory and ethics authorities in accordance with HHS regulations for the protection of human subjects (45 CFR §46.116 and §46.117) and Good Clinical Practice (ICH E6). Liver spheroids were formed as previously described.^[^ ^15^ ^]^ Briefly, PHH were seeded in 96‐well ultralow attachment (ULA) plates (Corning) with 1500 cells per well in 100 µL culture medium (Williams E medium supplemented with 2 mm l‐glutamine, 100 U mL^−1^ penicillin, 100 µg mL^−1^ streptomycin, 5.5 µg mL^−1^ transferrin, 6.7 ng mL^−1^ sodium selenite, 100 nm dexamethasone) with 10% fetal bovine serum. Unless indicated otherwise, liver spheroids were maintained in culture medium with 11 mm glucose and 0.1 nm insulin. Following aggregation, serum was phased out. For 2D PHH culture, cells were seeded at 3.5*10^5^ cells mL^−1^ in PHH culture medium onto 24‐well plates (Corning) coated with rat tail collagen I (Corning). After cells attached for 2 h, the medium was replaced with serum‐free PHH culture medium.

Primary human pancreatic islets were commercially acquired from Tissue Solutions (Glasgow, UK). Upon receipt, islets were allowed to recover for 48 h following the provider's instructions. Subsequently, cells were distributed in ULA plates (Corning) and maintained in serum‐free low glucose (3.5 mm) culture medium. Relevant demographic and medical information of all utilized liver and pancreas donors is provided in **Table** **1**.

**Table 1:** Demographic and medical information of the used tissue donors

Donor	Tissue	Age	Sex	Race	BMI	Cause of death	Hb1AC [%]
1	Liver	27	F	African American	28.2	Anoxia	N/A
2	Liver	30	F	Hispanic	30.8	Head trauma	N/A
3	Liver	25	M	Hispanic	32.2	Head trauma	N/A
4	Pancreas	30	F	Caucasian	34.2	Head trauma	5.7
5	Pancreas	26	M	Hispanic	26.4	Head trauma	5.4

BMI = body mass index; Hb1AC = glycated hemoglobin; N/A = not available.

### 2.6 Microphysiological Coculture Experiments

For microfluidic coculture experiments, devices were first coated with 120 µL (Biofloat Flex coating solution, faCellitate, Germany) for 3 min to avoid cell adhesion. Afterward, the solution was aspirated and chips were air dried for 30 min before loading opposite satellite chambers of the device with 25–30 liver spheroids and 10–20 primary human islets per experiment in Williams E medium with 11 mm or 3.5 mm glucose supplemented with 2 mm l‐glutamine, 100 U mL^−1^ penicillin, 100 µg mL^−1^ streptomycin, 5.5 µg mL^−1^ transferrin, 6.7 ng mL^−1^ sodium selenite, and 100 nm dexamethasone. Notably, the medium did not contain insulin by itself and, thus, any insulin found in the chips was released by the islets. Importantly, both liver spheroids and islets were loaded in insulin‐ and serum‐free culture medium. After loading of the devices within a sterile environment, the chips were closed, sealed and connected to a programmable pressure controller. To emulate postprandial pancreas–liver crosstalk, an alternating pumping and withdrawal program was initiated at a flow rate of 100 µL min^−1^ and total volumes of 85 µL per pumping‐withdrawal cycle for 4 h. Notably, the devices are compatible with isopropanol and UV sterilization protocols and can be reused multiple times without apparent changes in phenotypes of the cultured cells.

### 2.7 Viability Measurements

Viability of 3D cultures was evaluated by measuring ATP levels using the CellTiter‐Glo Luminescent Cell Viability Assay kit (Promega, Sweden). Briefly, PHH spheroids were incubated with the ATP reagent (provided in the kit) for 20 min at room temperature. Subsequently, luminescence was measured on a MicroBeta LumiJET 2460 Microplate Counter (Perkin Elmer, USA). On‐chip viability is expressed relative to freshly formed spheroids.

### 2.8 Metabolic Activity Measurements

Metabolic activity of liver spheroids was determined as previously described.^[^ ^33^ ^]^ In short, spheroids were exposed for 5 h to an established cocktail of 10 µm midazolam, 10 µm amodiaquine, 50 µm phenacetin, 10 µm diclofenac, and 5 µm dextromethorphan to quantify activity of CYP3A4, CYP2C8, CYP1A2, CYP2C9, and CYP2D6, respectively. Formation of the corresponding metabolites 1‐hydroxy midazolam, *N*‐desethyl amodiaquine, acetaminophen, 4‐hydroxy diclofenac, and dextrorphan were determined by LC‐MS/MS analysis with an AB Sciex API 6500+ triple quadrupole (AB Sciex LLC, MA, USA) coupled to a Waters Acquity I‐Class UPLC (Waters Corporation, MA, USA).

### 2.9 Gene Expression Profiling

Total RNA of liver spheroids and islets was isolated using the Zymo Mini Kit (R1054; Zymo Research). For the expression profiling of candidate genes, RNA was reverse transcribed into cDNA using SuperScript III Reverse Transcriptase (Invitrogen) and qPCRs were performed using the TaqMan probes provided in Table S1 of the Supporting Information. Expression levels were analyzed using the ΔΔCt method with GAPDH as reference gene.

### 2.10 Insulin Sensitivity Measurements

Insulin sensitivity of liver spheroids was measured by evaluating AKT phosphorylation using Western blotting. Liver spheroids were cultured for 7 days in culture medium supplemented with different insulin concentrations. Subsequently, spheroids were exposed for 10 min to high insulin levels (1.7 µm). 32–48 spheroids per sample were pooled and lysed in RIPA buffer supplemented with protease inhibitor (cOmplete Tablets EASYpack, Roche) and phosphatase inhibitor cocktail (PhosSTOP, Sigma). The membranes were blocked with 5% (w/v) bovine serum albumin (BSA) in TBS with 0.1% Tween‐20 for 1 h at room temperature and subsequently incubated overnight at 4 °C with antibodies against pAKT^Ser473^ (4060T, Cell Signaling Technology) and vinculin (129002, Abcam) as reference. Insulin sensitivity was measured by comparing the pAKT:vinculin intensity ratios before and after insulin stimulation.

### 2.11 Insulin Secretion Dynamics

To measure insulin secretion, primary human islets were starved by culture in low‐glucose culture medium (3.5 mm glucose) for at least 15 h. Subsequently, islets were transferred individually into new wells containing culture medium with postprandial glucose concentrations (11 mm). Supernatant was collected and islets were transferred serially into new wells multiple times. Insulin concentrations in the different supernatants were measured by ELISA (Mercodia).

### 2.12 Immunofluorescence

Primary human islets were fixed in 4% paraformaldehyde overnight then washed with PBS and incubated with 30% sucrose‐PBS at 4 °C until the microtissues sank. For cryo‐mount embedding, islets were first transferred into molds with OCT and were then frozen in an isopropanol dry ice bath. Subsequently, islets were sectioned at 8 µm thickness on a CryoStar NX70 cryostat (Epredia). For staining, sections were washed twice with PBS, blocked with PBTA buffer (5% BSA, 0.25% Triton X‐100, 0.01% NaN_3_ in PBS) for 2 h at room temperature and incubated overnight at 4 °C with monoclonal primary antibodies for insulin (1:500 rabbit‐*α*‐insulin; Cell Signaling), glucagon (1:1000 rabbit‐*α*‐glucagon; Cell Signaling) or somatostatin (1:1000 rat‐*α*‐somatostatin; BioRad). Subsequently, sections were washed 3 × 15 min with PBS at room temperature before adding the Alexa Fluor‐conjugated secondary antibody for 2 h at room temperature. After a final washing step, the slides were mounted using Prolong Gold Antifade mounting reagent with DAPI (Thermo Fisher). Images were obtained using a Zeiss LSM880 confocal microscope.

### 2.13 Immunohistochemistry

Liver spheroids were embedded in paraffin and 3 µm sections were stained using anti‐COL1A1 (ab34710, Abcam), anti‐CD68 (sc‐70761, Santa Cruz), and DAKO Real EnVision Detection System (Agilent) with peroxidase/DAB+ colorimetric detection, and Hämalaun counterstain. The stained slides were documented with an Olympus VS120 slide scanner with OlyVIA software v3.3 (Olympus).

### 2.14 RNA‐Sequencing

RNA was isolated as described above and samples were analyzed for integrity and abundance by capillary electrophoresis using the Fragment Analyzer System (Agilent). Subsequently, samples were processed using the NEBNext Ultra II Directional RNA Library Prep Kit (NEB #E7760S/L). Briefly, mRNA was isolated from total RNA by poly‐A capture using oligo‐dT magnetic beads. Subsequently, the mRNA was fragmented, and cDNA synthesis was performed. The cDNA was then ligated to the sequencing adapters and the resulting product was amplified by PCR. Clustering and DNA sequencing was performed on a NovaSeq6000 instrument (Illumina) using 1.1 nm of DNA input concentration at GenomeScan BV, Leiden, The Netherlands. Image analysis, base calling, and quality checks were performed using the RTA3.4.4 pipeline and Bcl2fastq (v2.20) conversion software (Illumina). Quality control, removal of genomic contaminants and ribosomal RNA, UMI‐based read deduplication, transcript assembly and quantification using Stringtie were conducted using the nf‐core/rnaseq.^[^ ^34^ ^]^ The raw data are available at the Gene Expression Omnibus (GEO) Archive with accession number GSE214764. Genes with an average number of fragments per kilo base per million mapped reads (FPKM) >0.5 across all samples were analyzed using Qlucore (Lund, Sweden). Significantly enriched pathways were identified based on the Reactome Pathway Database^[^ ^35^ ^]^ using the WebGestalt toolbox.^[^ ^36^ ^]^

### 2.15 Global Transcription Factor Activity Analysis

Activity profiles of 503 transcription factors were analyzed in primary human pancreatic islets in postprandial and fasting conditions using the ISMARA algorithm.^[^ ^37^ ^]^ Briefly, the activities of transcription factor motifs are inferred by fitting RNA‐Seq expression data into a linear model where a Gaussian prior on motif activities was used to avoid overfitting. The parameters of the prior distribution of the Gaussian likelihood model were estimated using fivefold cross‐validations. Transcription factor binding motifs were validated using human ChIP‐Seq data based on well‐curated annotations of promoters in JASPAR.^[^ ^38^ ^]^

### 2.16 Statistical Analyses

Results are presented as mean ± SEM unless stated otherwise. For determining statistical significance, unpaired two‐tailed heteroscedastic *t*‐tests were performed using GraphPad Prism with *n* ≥ 3 samples per group. All data are shown, and no outlier removal was performed. For all results, significance was defined as *p* ≤ 0.05.

## 3 Results

### 3.1 Design of a Pneumatically Actuated Microfluidic Coculture Device for the Investigation of Human Tissue Crosstalk

Ex vivo coculture of different human tissue models with physiological functionality constitutes an emerging strategy to understand the systems biology of tissue interactions. To accurately mimic organ‐to‐organ crosstalk, microfluidic devices should ideally fulfill multiple requirements. First, the setup should be compatible with long‐term on‐chip culture of organotypic tissue models, which requires stable pumping efficiency without significant evaporation, loss in pressure or the formation of gas bubbles. Second, the device should emulate tissue interactions not as a serial connection of different compartments but as a modular interconnected network in which signals from different tissues are integrated and redistributed thus enabling reciprocal crosstalk. Lastly, the chip setup should grant precise control over perfusion rates, ideally for each compartment separately, thereby allowing to adopt on‐chip perfusion kinetics to experimentally determined tissue‐specific in vivo parameters.

**Figure 1:**
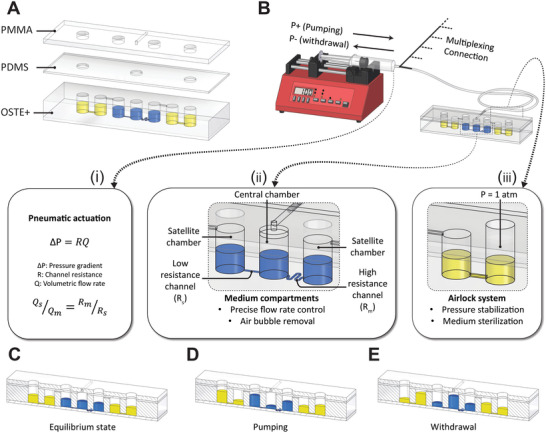
Schematic depiction of the pneumatically actuated microfluidic device design and its working principle. A) Hybrid microfluidic device comprised of off‐stoichiometry thiol–ene–epoxy (OSTE+), polydimethylsiloxane (PDMS), and polymethylmethacrylate (PMMA) materials. OSTE+ offers minimal drug absorption along with ease of manufacturability, PDMS allows gas permeability and PMMA provide a robust packaging. Note that the PDMS and PMMA layers are never in touch with the media in the device thus avoiding unfavorable drug absorption. B) After closing of the device the central chamber is connected to a pressure controller or syringe pump. Rhythmic application of alternating overpressure (pumping) and underpressure (withdrawal) to the central chamber enables controlled mass transport via pneumatic actuation (inset i). Stoichiometries of flow to the satellite chambers can be accurately controlled by modulating channel resistance (see methods; inset ii). The airlock systems maintain atmospheric pressure while avoiding contamination or evaporation (inset iii). C–E) Cross‐sectional images of the microfluidic device during actuation. (C) Upon loading of the central and satellite chambers with medium, the device is in equilibrium state. (D) Subsequently, application of pressure to the central chamber using pneumatic actuation causes the media to flow to the satellite chambers. (E) Following pumping, the gas is withdrawn resulting in a backward flow of media from the satellites to the central chamber.

To meet all these criteria, we thus designed a pneumatically actuated coculture platform comprised of a central chamber and multiple satellite chambers connected to the central chamber via µm‐sized fluidic channels. For ease of demonstration, an embodiment with two satellite chambers is shown (**Figure** **1**A). An analogous design with four satellites is shown in Figure S1A of the Supporting Information. For the scalable and high‐fidelity replication of microfluidic components including bottom and middle layers we used microreaction injection molding of OSTE+. Alternatively, we created hybrid microfluidic layers out of molded thiol–ene–epoxy and micromilled PMMA. To impart gas‐permeability to the system, a layer of PDMS was used on top of the fluidic layer. Importantly, the PDMS layer is never in direct contact with culture media, thereby avoiding issues pertaining to its absorption of hydrophobic peptides and small molecules.^[^ ^39^ ^]^ An additional PMMA layer with an l‐shaped fluidic channel connects the central chamber to a separate pressure controller unit. Importantly, both central and satellite chambers are filled with culture medium (≈40 µL per chamber in the equilibrium state; 120 µL total filling volume of the device) while leaving a gas pocket on top of the medium (Figure 1B).

The satellite chambers are connected to the central compartment by microfluidic channels with identical cross‐sections. By varying their length, we can precisely modulate channel resistance in accordance with Hagen–Poiseuille's law, thereby controlling the relative flow rates to the satellite chambers while only actuating the central compartment. If the pressure in all satellite chambers equals atmospheric pressure, the flow rates to the satellite chambers is controlled solely by the pressure, *P*, in the central chamber. The ratio of the average flow rate in the straight (*Q*_s_) and meandered (*Q*_m_) channels is inversely equal to their length

(7) $$Q_{s}/Q_{m}=R_{m}/R_{s}=L_{m}/L_{s}$$

Flow rates can thus be modulated by altering the resistance of the straight (*R*_s_) and meandering (*R*_m_) channels through their length (*L_s_* and *L*_m_). Such precise flow rate control is essential since coculture of different tissues necessitates exposure to shear stress of diverse magnitudes in perfused microfluidic devices, depending on the cell types.

A single pressure control unit introduces or retracts air into or out of the central chamber thereby modulating the gas pressure in the upper part of the central chamber. This pressure drives the fluid flow between central and satellite chambers (Figure 1C–E). As the pressure increases in the central chamber, the liquid medium flows through the microchannels into the satellite chambers. As a result, the liquid level in the satellite chambers increases, thus increasing the air pressure in the upper section of the chambers. To maintain atmospheric pressure, we introduced oil lock units. The motion of the oil allows to maintain atmospheric pressure in the satellite chambers while isolating the air trapped in each satellite, thus minimizing evaporation. The same principle applies when subsequently withdrawing air from the central chamber. Without oil locks, satellite air pocket pressure increases and requires dynamic increase of the applied pressure to maintain constant per‐cycle fluid displacement (Figure 1B, Supporting Information). Thus, alternating infusion and withdrawal of liquid through pneumatic actuation allows for the creation of a reciprocal flow between central and satellite chambers that in turn enables controlled mass transfer during tissue‐tissue coculture experiments.

### 3.2 The Pneumatically Actuated Microfluidic System Enables Efficient Mixing

**Figure 2:**
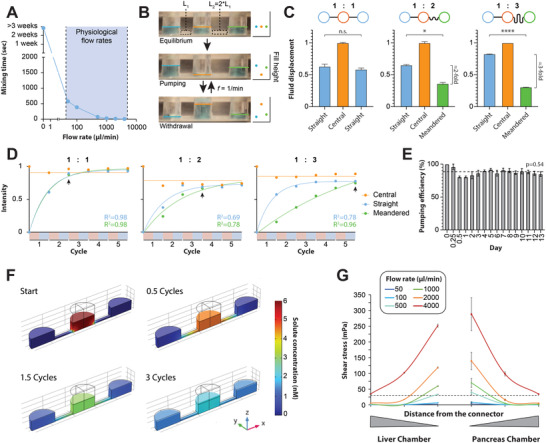
Pneumatic actuation achieves rapid and stable mixing across the entire range of physiologically relevant flow rates. A) Quantification of mixing kinetics. The indicated mixing times refer to the complete distribution of a colored marker from the central chamber to both satellite compartments. B) Side view of pneumatic actuation during equilibration, pumping, and withdrawal stages. Filling levels are indicated for the central (orange), left (blue), and right (green) chambers. The channel connecting the central chamber to the right compartment (L_2_) is twice as long as the connector with the left compartment (L_1_). During pumping, media is pressed from the central chamber to the side chambers with medium flowing back to the central chamber during the withdrawal stage. Note that the meandered chamber (green), is less filled and less emptied at the end of the pumping and withdrawal stages, respectively. C) Fluid displacement for the different compartments is shown for different channel ratios (left 1:1; middle 1:2; right 1:3). D) Mixing kinetics are shown for the different compartments during different pumping cycle (light red = pumping phase; light blue = withdrawal phase). Arrows indicate the cycle after which complete mixing was achieved. Note that increasing channel ratios result in reduced fluid displacement and increased mixing times in the meandered channel. E) Pumping efficiency remains stable over at least 13 days (*p* = 0.54 for slope deviation from zero; F‐test). Pumping efficiency is indicated as fluid displacement at the indicated days relative to fluid displacement at the start of the experiment. F) Snapshots of the simulation results of solute distribution in the chip at different cycle phases. Note that local solute concentrations are almost equilibrated after 3 cycles. G) The maximum wall shear stress on the cell surface of liver spheroids (left) and pancreatic islets (right) as a function of distance from the connecting channels is shown. Shear stress increases with flow rate and proximity to the connector. Flow rates ≤100 µL min^−1^ maintain physiological shear stress ≤30 mPa. Error bars indicate SEM. *, **, ***, and **** indicate *p* < 0.05, *p* < 0.01, *p* < 0.001, and *p* < 0.0001 based on heteroscedastic two‐tailed *t*‐tests, respectively.

Perfusion rates of different human tissues can differ around 40‐fold in vivo with flow estimates ranging between 5 mL/100 g min^−1^ in subcutaneous adipose tissue^[^ ^40^ ^]^ to 35 mL/100 g min^−1^ in pancreas,^[^ ^41^ ^]^ 100 mL/100 g min^−1^ in liver^[^ ^42^ ^]^ and up to 200 mL/100 g min^−1^ in skeletal muscle during exercise.^[^ ^43^, ^44^ ^]^ Devices for the ex vivo culture of human tissues should thus allow for accurate control of flow and mixing dynamics across a wide range of flow rates. We therefore evaluated mixing dynamics in the device across different flow rates from 0 to 4000 µL min^−1^ (**Figure** **2**A). Without perfusion (static culture), diffusion alone was not sufficient to achieve mixing even after 3 weeks. By contrast, complete mixing was observed upon activation of perfusion within 2–4 pumping–withdrawal cycles at any flow rate tested within <10 min.

Next, we experimentally tested the effects of modulating the resistance of the microfluidic channels that link the central chamber and pumping unit to the satellite chambers (Figure 2B,C). To this end, we developed a real‐time camera setup that recorded and longitudinally quantified fluid displacement in both central and satellite chambers. As expected, when the lengths of the connecting channels of both satellite chambers were the same, fluid displacement in both satellite chambers was identical (*p* = 0.5). When lengths were varied between the satellites by 1:2 or 1:3, significant differences in per‐cycle fluid displacement were observed between the chambers (*p* < 0.05).

Fast and homogenous mixing is crucial to achieve efficient mass transport between compartments. In the presented system, both the pumping and withdrawal phase contribute to the homogenization within the device. However, in chips with meandered channels, the higher‐resistance compartments are only partially emptied during withdrawal, which increases the times required for complete mixing. To assess the magnitude of this effect, we directly quantified the impact of altered channel resistance on mixing kinetics (Figure 2D). Notably, we found that mixing time increased in the meandered channel with increasing channel‐length ratios; however, even in chips with a 1:3 channel ratio, complete mixing was achieved in both compartments after ≤5 cycles (corresponding to ≈500 s).

To test the stability of the device, chips were subjected to one pumping cycle per 110 s over the course of 13 days, corresponding to ≈11 000 pumping cycles. Importantly, fluid displacement per pumping cycle remained constant and no significant decrease in pumping efficiency was evident (Figure 2E), indicating that the tested devices remained tight without leakage of gas or fluid and that channels did not clog due to air bubbles, which constitutes a common problem in many microfluidic devices. Furthermore, devices remain leak‐tight even after being reused many times over multiple years.

Shear stress constitutes an important factor that can influence cell function. To adopt flow rates that generate shear close to in vivo values, we used physics‐based modeling and simulation. We first established a model in COMSOL based on FEM that solves transient partial differential equations of linear momentum, mass transport, and diffusion (Figure 2F). Notably, the model closely recapitulated fluid displacement and mixing kinetics of the fabricated devices (Figure S2, Supporting Information). We then used the model to simulate shear stress for liver spheroids and primary human islets in the satellite compartments as a function of flow rate. For liver spheroids we assumed sizes of 200 µm,^[^ ^21^ ^]^ whereas islet sizes are more heterogeneous and typically vary between 50 and 300 µm.^[^ ^45^ ^]^ As expected, for all microtissue sizes shear stress increased with flow rates and shear was highest for microtissues close to the channels connecting the satellite chambers to the central compartment (Figure 2G). At flow rates of 100 µL min^−1^ we found that the maximal shear was around 30 mPa for liver spheroids, which corresponds to physiological liver shear forces.^[^ ^46^ ^]^ For islets, shear stress was overall higher due to their variation in size and only flow rates of 50 µL min^−1^ did not exceed physiological shear. Culturing liver and pancreatic islets in chips with meandered channels (1:2) thus allowed us to achieve mass transfer at physiologically relevant tissue‐specific shear using a single pneumatic actuation unit.

Combined, these results confirm that it is possible to achieve heterologous mass transport through homologous pumping by introducing microfluidic meanders. Furthermore, they show that efficient mixing at physiologically relevant flow rates is achieved in the device within minutes, resembling in vivo distribution kinetics of glucose or intravenously administered drugs.

### 3.3 Integrated On‐Chip Profiling of Primary Human Islets Reveals Molecular Glucose Response Signatures in Normo‐ and Prediabetic Hyperglycemia

**Figure 3:**
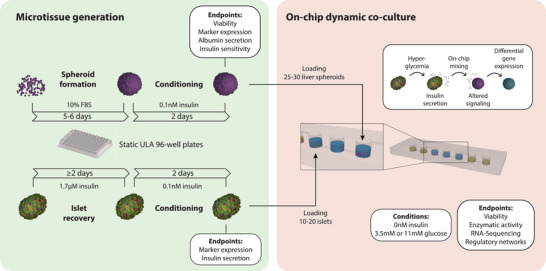
Schematic depiction of the experimental workflow. Pancreatic islets and liver spheroids are generated and recovered in ultralow attachment (ULA) plates. After conditioning for two days to low (0.1 nm) insulin, the microtissues are washed and transferred into the pneumatically actuated coculture device in media without insulin. In the integrated device, islets and liver spheroids are cultured in either low (3.5 mm) or high (11 mm) glucose concentrations and molecular and functional responses are evaluated using RNA‐Sequencing and regulatory network decomposition.

**Figure 4:**
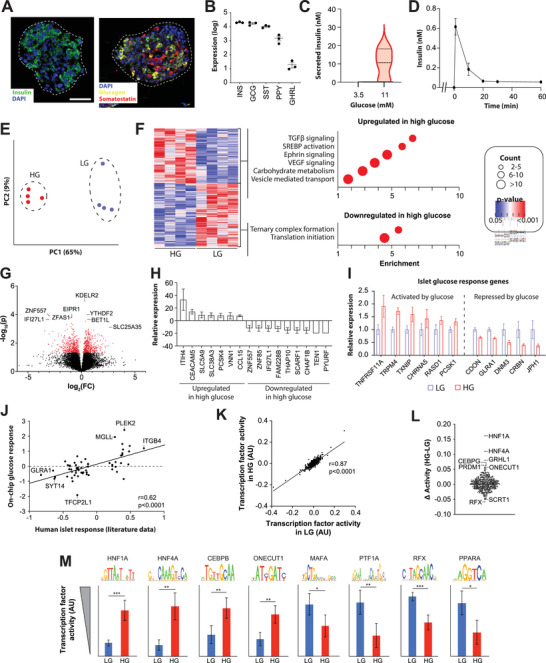
On‐chip exposure of primary human islets to hyperglycemia results in *β*‐cell stress, dedifferentiation and islet exhaustion. A) Immunofluorescent staining of primary human islets for insulin (*β*‐cells), glucagon (*α*‐cells), and somatostatin (*δ*‐cells). Dashed line indicates island circumference. Scale bar = 100 µm. B) Expression levels of endocrine hormones secreted by *α*‐cells (GCG), *β*‐cells (INS), *δ*‐cells (SST), *γ*‐cells (PPY), and *ε*‐cells (GHRL). Expression is shown as log_10_(FPKM) and sorted in descending order. *N* = 3. **C)** Quantification of insulin secretion after exposure of islets to hypoglycemic (3.5 mm glucose) or hyperglycemic (11 mm glucose) conditions. For each measurement, 10–20 islets were exposed to the indicated glucose concentrations for 4 h in 120 µL. *N* = 3. D) Insulin secretion dynamics show that insulin is secreted very rapidly with the bulk of insulin release being detected within the first few minutes after exposure to an increase in glucose concentrations from 3.5 to 11 mm. *N* = 4. E) Principal component analysis of RNA‐Sequencing data show that isogenic islets exposed to low glucose (LG; 3.5 mm) and high glucose (HG; 11 mm) conditions rapidly alter their transcriptomic signatures. F) Left: heatmap visualization of differentially expressed genes. Right: dot plot visualization of Reactome pathway analysis of the indicated clusters. Dot size indicates the number of pathway‐associated genes in the respective cluster; dot color indicates the statistical strength of the association. G) Volcano plot showing the changes in gene expression expressed as fold‐change (FC) of high glucose compared to low glucose. H) The most highly up‐ and downregulated genes include various metabolic modulators, such as the glucose transporter *SLC5A9* or the lipid regulator *PCSK4*, as well as the proinflammatory chemokine *CCL15*. I) The on‐chip glucose response closely resembles response signatures of primary human islets based on established candidate markers. J) Comparison of differentially expressed genes identified in chronic hyperglycemia^[^ ^49^ ^]^ with our chip data indicates a clear correlation. K) Scatter plot showing the correlation between the motif activities of 503 transcription factors in high glucose (HG) and low glucose (LG) conditions. L) Transcription factors with the largest differences in motif activities between glucose levels are indicated. M) Activity of candidate transcription factors with known importance in islet function. Note that activity signatures of transcription factors involved in *β*‐cell stress are induced while motifs associated with pancreatic differentiation are overall decreased. Sequence logos show the binding motif of the respective transcription factor. RNA‐Seq data was analyzed from *N* = 4 biological replicates. Error bars indicate SEM. *, **, and *** indicate *p* < 0.05, *p* < 0.01, and *p* < 0.001 based on heteroscedastic two‐tailed *t*‐tests, respectively.

We utilized the setup to study the metabolic interaction between primary human liver spheroids and pancreatic islets. To this end, islets and liver spheroids were first generated and conditioned in static ULA plates before being transferred into the microfluidic chips for dynamic coculture experiments (**Figure** **3**). The islets were derived from patients undergoing pancreatectomy and comprised *α*‐, *β*‐, and *δ*‐cells, as evident by expression of their canonical markers glucagon, insulin, and somatostatin, respectively (**Figure** **4**A). Furthermore, the presence of other minor endocrine cell types, such as *γ*‐ and *ε*‐cells was evident, was confirmed by expression of the respective marker genes *PPY* and *GHRL*, respectively (Figure 4B). To test functionality, intact islets were exposed to glucose levels resembling healthy (3.5 mm) and prediabetic (11 mm) concentrations. Notably, while islets secreted almost no insulin in fasting conditions (27 pm ± 9 pm), they rapidly secreted insulin upon glucose challenge, reaching levels of 9.6 ± 4.2 nm after 4 h, resembling prediabetic hyperinsulinemia (Figure 4C). Insulin was secreted almost immediately upon exposure to high glucose with the highest secretion during the first 3 min, closely resembling in vivo insulin release patterns after a meal (Figure 4D).

We then evaluated the molecular glucose response signatures of pancreatic islets in coculture with liver spheroids in opposing satellite chambers of the fabricated OSTE chips. Notably, exposure to elevated glucose levels resulted in significant transcriptomic differences with 840 differentially expressed genes (Figure 4E; Table S2, Supporting Information). Among the significantly activated pathways in hyperglycemic conditions were TGF*β* signaling (*p* = 0.0002), SREBP activation (*p* = 0.0002), as well as carbohydrate metabolism (*p* = 0.00004) and vesicle‐mediated transport (*p* = 0.0007; Figure 4F). By contrast, ternary complex formation (*p* = 0.00009) and translation initiation (*p* = 1*10^−6^) were highly downregulated, hallmarked by the reduction of initiation factors and various ribosomal proteins. These findings are consistent with previous reports indicating that activation of the UPR due to endoplasmic reticulum (ER) stress^[^ ^47^ ^]^ or high fat diet^[^ ^48^ ^]^ restricts translation in islet in vivo.

Consistent with these observations, we found that the KDEL receptor *KDELR2*, which is activated during UPR,^[^ ^49^ ^]^ was the most significantly upregulated gene in islets cultured in the integrated chip upon exposure to a glucose bolus (Figure 4G). Further significantly increased genes included the mitochondrial dicarboxylate carrier *SLC25A35*, as well as *YTHDF2*, encoding a reader of the *N* ^6^‐methyladenosine (m^6^A) RNA modification, which is essential for *β*‐cell function.^[^ ^50^ ^]^ The most significantly downregulated gene was *EIPR1*, which has been shown to be involved in the maturation of insulin secretory granules,^[^ ^51^ ^]^ suggesting a negative feedback mechanism to render islets transiently refractory to glucose‐induced insulin secretion. Among the most strongly upregulated genes in islets were *ITIH4*, *CEACAM5*, *SLC5A9*, *PCSK4*, and *CCL15*, whereas *TEN1*, *PYURF*, and *CHAF1B* were most downregulated (Figure 4H).

Next, we compared on‐chip islet expression of candidate glucose response genes to literature data from intact human islets.^[^ ^52^, ^53^ ^]^ In agreement with these data, the canonical glucose response genes *TNFRSF11A*, *TRPM4*, *TXNIP*, *CHRNA5*, *RASD1*, and *PCSK1* were upregulated in prediabetic glucose concentrations while expression of *CDON*, *GLRA1*, *DNM3*, *CRBN*, and *JPH1* were repressed by glucose (Figure 4I). Furthermore, systematic comparison of expression alterations across all genes found to be differentially expressed in chronic hyperglycemia^[^ ^52^ ^]^ with our chip data, demonstrated a highly significant correlation (*r* = 0.62; *p* < 0.0001; Figure 4J). These results demonstrate that acute metabolic responses of islets to a physiological glucose bolus on‐chip recapitulate overall gene expression alterations observed in chronic hyperglycemia, thus providing an appealing tool for the multiplexed dynamic monitoring of glycemic control of different tissues in an integrated circuit.

To explore the underlying molecular events controlling the islet response to glucose, we conducted a comparative global analysis of the activities of 503 transcription factors by statistically associating experimentally determined transcription factor binding motifs in gene promoters with their downstream transcriptional regulation. As expected, we find the transcription factor activities are overall highly correlated between glucose concentrations (*r* = 0.87; *p* < 0.0001; Figure 4K; Table S3, Supporting Information). However, individual transcription factors showed significant differences in motif activities. Specifically, activities of HNF1A and HNF4A were highly increased in hyperglycemia (Figure 4L,M). Both transcription factors are required for glucose‐stimulated insulin secretion and normal exocrine differentiation,^[^ ^54^, ^55^ ^]^ and genetic variations in both genes have been associated with maturity‐onset diabetes of the young.^[^ ^56^ ^]^ In addition, CEBPB was significantly activated, which has been shown to contribute to *β*‐cell failure in mice by causing a disequilibrium between ER protein load and its folding capacity,^[^ ^57^ ^]^ thereby providing a molecular link between hyperglycemia, ER stress, and the UPR.

Further transcription factors with significantly altered activity included ONECUT1 and MAFA, whose activities were increased and decreased in hyperglycemic conditions, respectively. Interestingly, *ONECUT1*, a genetic risk gene for monogenic recessive diabetes,^[^ ^58^ ^]^ has been shown to directly repress expression of *MAFA*,^[^ ^59^ ^]^ the key regulator of insulin expression in *β*‐cells,^[^ ^60^ ^]^ suggesting that the regulatory network that underlies insulin control in vivo is maintained on‐chip. Among the transcription factors whose activities were found to be most downregulated were the pancreatic differentiation PTF1A, which is required for pancreatic specification,^[^ ^61^ ^]^ as well as RFX transcription factors that are critical for the maintenance of pancreatic identity.^[^ ^62^ ^]^ Furthermore, PPARA activity was significantly repressed in prediabetic glucose conditions, in agreement with previous in vitro and in vivo results.^[^ ^63^, ^64^ ^]^ Combined, these results indicate that a high glucose bolus rapidly increases *β*‐cell stress, characterized by increased TGF*β* signaling, pancreatic dedifferentiation, and a recapitulation of the molecular hallmarks of UPR.

### 3.4 Functional Metabolic Crosstalk of Organotypic Primary Human Liver Spheroids and Pancreatic Islets Uncover Tissue‐Specific Metabolic Regulatory Networks

Next, we evaluated whether we could detect functional tissue communication in the coculture device along the pancreas–liver axis. To this end, we first evaluated the on‐chip viability and molecular phenotypes of 3D cultured primary human liver spheroids in monoculture. Liver spheroids showed smooth peripheries and were positive for bona fide hepatocyte markers, such as CYP3A4 and ALB (Figure S3A,B, Supporting Information), as previously reported.^[^ ^15^ ^]^ Besides hepatocytes, spheroids also contained low levels of Kupffer cells, as evident by individual CD68 positive cells (Figure S3C, Supporting Information). Notably, the pattern of COL1A1 expression was diffuse and overall low in liver spheroids in agreement with the histological assessment of nonfibrotic human liver sections (Figure S3D, Supporting Information).

**Figure 5:**
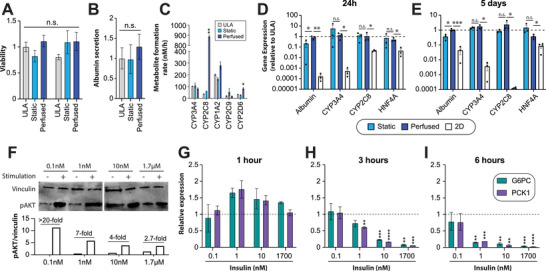
Liver spheroids retain mature hepatic phenotypes and remain insulin sensitive on‐chip. All panels show data from liver spheroid monocultures. A) Viability of 3D primary human liver cultures remained stable for at least five days and was similar to established gold‐standard culture of isogenic spheroids in ultralow attachment (ULA) plates. *N* = 3–13 spheroids per group. B) Liver spheroids retained hepatic functionality on‐chip as evidenced by stable albumin secretion rates. *N* = 3 replicate experiments from three pooled spheroids each. C) Metabolic cytochrome P450 (CYP) activity of liver spheroids is stimulated in perfused devices. D,E) Expression levels of the hepatic genes *ALB*, *CYP3A4*, *CYP2C8*, and *HNF4A* remained stable after 24 h (D) and 5 days (E) of on‐chip culture compared to 3D liver spheroids on ULA plates and were significantly higher than in isogenic 2D monolayers. *N* = 3. F) Hepatic insulin sensitivity was profiled by Western blot against phosphorylated AKT (pAKT) using vinculin as loading control. Cells were cultured in the indicated insulin concentrations for 7 days and pAKT levels were evaluated 10 min after stimulation with high (1.7 µm) insulin. Note that insulin sensitivity decreases with increasing insulin levels during maintenance culture. Insulin response dynamics were evaluated in insulin sensitive liver spheroids 1 h (G), 3 h (H), and 6 h (I) after stimulation with the indicated insulin concentrations by profiling expression of *G6PC* and *PCK1*, genes known to be repressed by insulin in vivo. *N* = 3. Error bars indicate SEM. *, **, and *** indicate *p* < 0.05, *p* < 0.01, and *p* < 0.001 based on heteroscedastic two‐tailed *t*‐tests, respectively. n.s. = not significant; i.e., *p* > 0.05.

In our OSTE devices, liver spheroids remained viable and functional, as evidenced by high and stable ATP levels, as well as albumin secretion rates and metabolic activity that were unchanged or even higher than in ULA cultures (Figure 4A–C). Since primary human hepatocytes are known to rapidly dedifferentiate in inappropriate culture conditions within few hours,^[^ ^13^ ^]^ we evaluated expression levels of key hepatic genes (*ALB*, *CYP3A4*, *CYP2C8*, and *HNF4A*) in spheroid cultures after 24 h and 5 days of on‐chip culture. Marker expression of on‐chip 3D liver spheroids was overall similar compared to spheroid cultures in static ULA plates (**Figure** **5**D,E). Perfusion significantly improved ALB expression but did not have marked effect on expression of the other hepatic markers. By contrast, on‐chip 3D liver spheroid cultures featured expression levels of all tested markers that between 5‐fold and >1000‐fold higher than isogenic 2D monolayers (Figure 5D,E). Combined, these results indicate that the device supports the stable maintenance of physiologically relevant liver phenotypes of 3D liver spheroids in perfused conditions.

We then profiled insulin response profiles in liver spheroids by evaluating AKT phosphorylation (Figure 5F). Notably, when spheroids were cultured in conventional culture media containing 1.7 µm insulin, only a minor 2.7‐fold increase in AKT phosphorylation was detectable 10 min after insulin stimulation. When maintenance insulin levels were however decreased, the magnitude of response increased drastically to >20‐fold. We thus used 0.1 nm insulin for the establishment of 3D liver cultures prior to loading into the coculture chip for all further experiments. To assess insulin response dynamics, we exposed liver spheroids to defined concentrations of insulin (Figure 5G–I). Importantly, *G6PC* and *PCK1*, key gluconeogenetic genes that are repressed by insulin in vivo,^[^ ^65^ ^]^ were significantly and dose‐dependently downregulated at insulin concentrations ≥1 nm as early as 3 h, whereas expression patterns between the two genes became more divergent after 6 h. Based on these results, we further comprehensively profiled the acute molecular response of human liver spheroids in a self‐regulated coculture with pancreatic islets after 4 h using RNA‐Seq, importantly, without the addition of extrinsic insulin.

**Figure 6:**
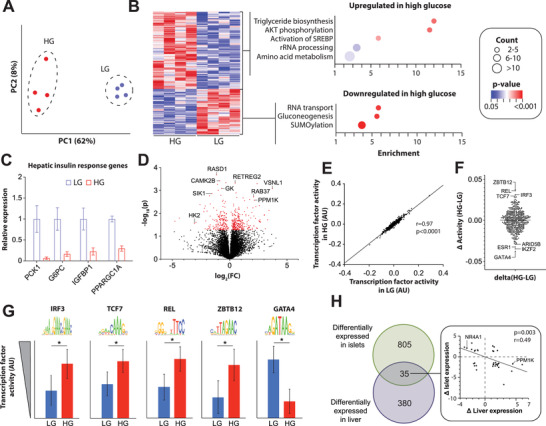
Profiling of 3D primary human liver cultures reveal the counter‐regulation of transcriptional programs in liver and pancreas. All panels show analyses of liver spheroids cocultured on‐chip together with pancreatic islets. A) Principal component analysis of RNA‐Sequencing data shows that liver spheroids in the integrated coculture chip undergo rapid molecular changes upon glucose challenge. B) Left: heatmap visualization of differentially expressed genes. Right: dot plot visualization of Reactome pathway analysis of the indicated clusters. Dot size indicates the number of pathway‐associated genes in the respective cluster; dot color indicates the statistical strength of the association. Note that pathways downstream of insulin signaling (AKT phosphorylation, activation of SREBP) are activated in high glucose, demonstrating functional interaction between the tissues. C) The canonical insulin response markers *PCK1*, *G6PC*, *IGFBP1*, and *PPARGC1A* (PGC1*α*) are repressed on‐chip after exposure to prediabetic (11 mm) glucose levels. D) Volcano plot showing the changes in gene expression expressed as fold‐change (FC) of high glucose compared to low glucose. E) Scatter plot showing the correlation between the motif activities of 503 transcription factors in liver spheroids in high glucose (HG) and low glucose (LG) conditions. F) Transcription factors with the largest differences in motif activities between glucose levels are indicated. G) Activity of candidate transcription factors is shown. Note that transcription factors implicated in the hepatic insulin resistance are significantly increased in HG. Sequence logos show the binding motif of the respective transcription factor. H) Venn diagram showing the overlap of differentially expressed genes in liver and islet after high glucose exposure. Interestingly, the overlap is relatively small and in significant anticorrelation (*p* = 0.003; F‐test), indicative of the tissue‐specificity of the human metabolic response signatures. Experiments were done with *N* = 4 biological replicates. Error bars indicate SEM. * indicate *p* < 0.05 based on heteroscedastic two‐tailed *t*‐tests.

Exposure of the integrated device to elevated glucose levels resulted in significant transcriptomic differences in the liver compartment (**Figure** **6**A; Table S4, Supporting Information). Pathway analyses of differentially expressed genes indicated that anabolic responses, including triglyceride biosynthesis (*p* = 0.01), SREBP activation (*p* = 0.02), and amino acid metabolism (*p* = 0.04) were significantly increased while glucose catabolism was decreased (*p* = 0.003; Figure 6B). Furthermore, AKT phosphorylation was significantly increased (*p* = 0.01), indicating intracellular activation of insulin signaling in hepatocytes. To further corroborate the effect of glucose on pancreas–liver crosstalk, we evaluated the expression of canonical hepatic insulin response genes (Figure 6C). Importantly, we found that *PCK1* and *G6PC* were downregulated by 16‐fold and 6‐fold upon glucose challenge, respectively, compared to isogenic coculture chips in low glucose. Similarly, *IGFBP1* and *PPARGC1A* (encoding PGC1*α*) known to be repressed by insulin in the liver in vivo,^[^ ^66^ ^]^ were found to be significantly downregulated in liver spheroids. Thus, elevation of glucose concentrations to postprandial levels in a liver–pancreas coculture chip result in the repression of insulin response genes in the liver compartment without addition of extrinsic insulin, demonstrating the functional communication between these primary human 3D organotypic cultures.

Besides these well‐established markers we identified a total of 415 genes that were differentially expressed in cocultured 3D primary human liver spheroids. Among the most significantly upregulated genes upon glucose exposure in the liver were the Ca2+ sensor protein *VSNL1*, the GTPase *RAB37* and *RETREG2* while *RASD1*, *CAMK2B*, and glycerol kinase (*GK*) were most highly downregulated (Figure 6D). To identify putative transducers of these alterations, we again conducted ISMARA analyses. The magnitude of responses in liver spheroids was overall lower than in pancreatic islets, as evident by a closer agreement of transcription factor activities in low glucose and high glucose conditions (Figure 6E; *r* = 0.97 in liver compared to *r* = 0.87 in islets). However, multiple transcription factors with significantly altered activity profiles were identified (Table S5, Supporting Information). Among the most strongly affected transcription factors was IRF3, whose activity significantly increased in high glucose upon coculture with pancreatic islets (Figure 6F,G). IRF3 has been shown to be upregulated in NAFLD in humans and IRF3 has been suggested as a critical causal mediator linking obesity‐induced inflammation and dysglycemia.^[^ ^67^ ^]^ Further significant factors include the Wnt/*β*‐catenin signaling cofactor TCF7 that has been strongly implicated in the control of hepatic gluconeogenesis,^[^ ^68^, ^69^ ^]^ and REL, a key effector of NF*κ*B signaling whose upregulation promotes insulin resistance.^[^ ^70^ ^]^

Interestingly, of all differentially expressed genes, <10% overlapped between liver spheroids and islets in the coculture chips (Figure 6H). Furthermore, among those, the direction of response was in significant anticorrelation (*r* = −0.49; *p* = 0.003). For instance, *PPM1K* was 4.5‐fold upregulated in high glucose in the liver compartment, while it was downregulated 1.7‐fold in islets (both *p* = 0.03). In the islet, PPM1K has been implicated in the control of insulin expression^[^ ^71^ ^]^ and its downregulation is in line with other aforementioned negative feedback loops that inhibit insulin translation following hyperglycemic stress to relieve the ER folding load. By contrast, in the liver, PPM1K controls the rate‐limiting step of BCAA catabolism, but has only shown to be repressed by fructose, not by glucose.^[^ ^72^ ^]^ Inversely, the orphan nuclear receptor NR4A1 is repressed 3.3‐fold in our on‐chip liver spheroids (*p* = 0.04), whereas it is induced 2.3‐fold in islets (*p* = 0.03). In the liver, NR4A1 counteracts lipogenesis by inhibiting SREBP,^[^ ^73^ ^]^ whereas NR4A1 in islets protects *β*‐cells from ER stress^[^ ^74^ ^]^ and controls islet proliferation.^[^ ^75^ ^]^ Combined, these results suggest a surprisingly large counter‐regulation of transcriptional programs in liver and pancreas and demonstrate the importance of considering tissue‐specific regulatory networks for understanding systemic responses to metabolic cues.

## 4 Discussion

Microphysiological tissue models have been hailed as promising prospects to improve preclinical‐to‐clinical result translation and break the long‐standing trend of declining drug approval rates per inflation‐adjusted research spending.^[^ ^76^, ^77^, ^78^ ^]^ While countless single tissue models provide evidence of improved cellular morphology, phenotype or functionality, the number of multitissue models is, while growing, still relatively small. Previously presented constellations primarily focus on applications related to pharmacokinetics or drug toxicity, involving cocultures of liver and kidney,^[^ ^79^, ^80^ ^]^ liver and heart^[^ ^81^, ^82^ ^]^ or liver and intestine.^[^ ^83^, ^84^, ^85^ ^]^ In addition, various systems have been presented to model physiological functions including gut–brain crosstalk^[^ ^86^, ^87^ ^]^ or glycemic control.^[^ ^25^, ^26^, ^88^ ^]^ Particularly for the latter, molecular and metabolic phenotypes that closely resemble their in vivo counterparts are essential to allow accurate interpretation of increasingly complex results. However, to the best of our knowledge, no microphysiological coculture systems have yet been presented that study the interaction between organotypic culture models of primary human cells. The work presented here thus constitutes the first report of crosstalk between 3D human tissue models ex vivo and provides a comprehensive overview of the network biology of glycemic control.

To facilitate model acceptance and dissemination for pharmacological and toxicological applications, it is of paramount importance to align with drug developer requirements and needs. This entails that the device should be fabricated from biocompatible materials with minimal absorption of drugs and hormones^[^ ^89^, ^90^ ^]^ or leaching of uncrosslinked oligomers.^[^ ^91^ ^]^ Furthermore, fabrication should be able to provide sufficient of numbers of devices for preclinical testing. Here, we used RIM of thiol–ene–epoxy polymers, which allows for rapid transitioning from academic prototyping to scalable fabrication,^[^ ^30^, ^31^ ^]^ thus opening possibilities for the wider dissemination within translational pharmacology.

The utilization of cell models to infer in vivo metabolic effects is challenging due to drastic differences in cellular microenvironments. Major difficulties for result translation stem from drastically different exposure of cells to nutrients and hormones compared between in vitro and in vivo. For instance, while physiological insulin concentrations pivot between 0.05 nm (fasting) and 2 nm (postprandial), commonly used culture media contain 1.7 µm insulin resulting in insulin resistance and altered metabolic configurations.^[^ ^92^ ^]^ Importantly, insulin resistance caused by excessive insulin exposure is long‐lasting, calling into question metabolic results obtained using cell lines that have been propagated in high‐insulin media for dozens of passages, even if the cells at the time of studies are cultured at low insulin concentrations. Similarly, physiological glucose levels fluctuate between 3.5 mm (fasting) and 7.5 mm (postprandial), but can reach 11 mm in prediabetes and up to 20 mm in clinically manifest diabetes; however, some previous studies used glucose concentrations up to 50 mm to measure glucose consumption rates.^[^ ^93^ ^]^ Importantly, we previously established that the culture conditions used in this work result in in vitro glucose consumption dynamics in response to insulin that closely resemble glucose uptake in humans measured by positron emission tomography (liver spheroids in vitro: 6.1 ± 1.7 fmol*cell^−1^*h^−1^, human liver in vivo: 4.2 to 31.8 fmol*cell^−1^*h^−1^), assuring that the model emulates metabolic features in vivo.^[^ ^21^ ^]^ Furthermore, we scaled the number of islets per device to reach physiological insulin concentrations after glucose challenge. Combined with physiological perfusion rates in the device that allow for complete mixing ≈5–10 min, these parameters result in nutrient and hormone exposure patterns upon exposure to a glucose bolus that accurately resemble in vivo patterns after a meal.

The use of a microfluidic device that interfaces 3D organotypic culture models offers new possibilities to study and functionally modulate human tissue interactions. However, this capability comes at the cost of increasing complexity. Liver spheroids can be generated with highly reproducible sizes of 220 ± 16 µm S.D. in diameter using cryopreserved hepatocytes,^[^ ^21^ ^]^ which allows to use cells from the same donors for replicate experiments, thereby increases experimental reproducibility. By contrast, pancreatic islets are highly heterogeneous in size and cannot be cryopreserved for future use in tissue culture. While initial testing of islet functionality using glucose challenge and the use of multiple islets per chip (here, we used 10–20) can buffer this variability to some extent, islet‐to‐islet heterogeneity can nevertheless increase interexperimental variability. Due to this increased complexity, microfluidic systems that use primary human tissues are more suitable for mechanistic investigation and in‐depth testing at the hit‐to‐lead or lead optimization stages, rather than for early‐stage high‐throughput screening or hit triaging.

**Figure 7:**
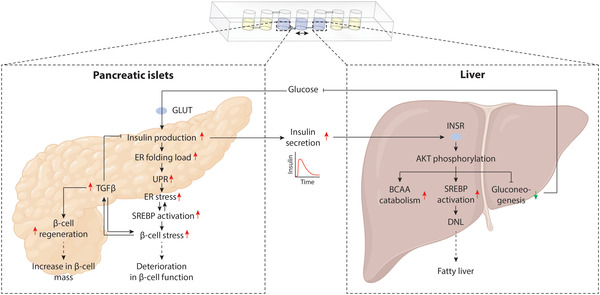
Schematic depiction of the interacting molecular networks in prediabetic hyperglycemia. By integrating comprehensive transcriptomic profiling with global transcription factor motif activity analyses and functional endpoints, we show that pathological glucose challenge causes insulin secretion, which entails an unfolded protein response (UPR) and endoplasmic reticulum (ER) stress. Compounded by activation of SREBP activation, these molecular alterations result in *β*‐cell stress and align with the observed deterioration in *β*‐cell function observed in diabetic individuals in vivo. Notably, we identify significant activation of TGF*β* signaling, both at the level of the ligand and downstream transducers, which is suggested to act as a negative feedback mechanism to limit further insulin expression and thus mitigate ER stress. Furthermore, our data indicate the activation of islet regenerative programs providing a molecular link between hyperglycemia and the increase in *β*‐cell mass in diabetes. In the liver, we find that the elevated insulin levels activate the insulin receptor (INSR), resulting in the phosphorylation of the central insulin signaling transducer AKT and the activation of de novo lipogenesis (DNL), branched chain amino acid (BCAA) catabolism as well as the pronounced inhibition of gluconeogenesis.

Critically, our results demonstrate that glucose challenge of the integrated device rapidly resulted in key metabolic endpoints, such as hormone secretion dynamics, AKT phosphorylation and repression of gluconeogenesis that closely captured in vivo responses (**Figure** **7**). Furthermore, using a systems biology approach to analyze the network biology of islet–liver interactions upon glucose challenge, we identified that exposure of islets from nondiabetic donors to prediabetic glucose levels (11 mm) resulted in a highly significant increase in ER stress. Upon elevations in glucose levels, *β*‐cells initiate the synthesis of around 10^6^ proinsulin molecules per minute resulting in a drastic increase in protein folding load in the ER and an activation of the UPR.^[^ ^94^ ^]^ Notably, the canonical UPR gene *KDELR2* that is known to be upregulated by chemical or genetic induction of ER stress^[^ ^95^ ^]^ was the most significantly activated gene in prediabetic glucose concentrations in our data. Transcriptomic analyses furthermore identified activation of SREBP and induction of TGF*β* signaling as significant responses to hyperglycemia. In mice, SREBP has been identified as a critical mediator of *β*‐cell glucotoxicity in chronic hyperglycemia^[^ ^96^ ^]^ and recent work in cell lines provided evidence for a positive feedback loop between ER stress, UPR and SREBP induction mediated by PERK.^[^ ^97^ ^]^ In contrast to SREBP, TGF*β* signaling has been implicated in the inhibition of MAFA^[^ ^98^ ^]^ and downstream insulin production,^[^ ^99^ ^]^ as well as the initiation of islet proliferation,^[^ ^100^, ^101^ ^]^ aligning with the results of our transcription factor activity analyses that indicate a coordinated downregulation of MAFA, PTF1A, and RFX. Combined, our results indicate that exposure to prediabetic glucose concentrations rapidly induce molecular programs of *β*‐cell stress and that TGF*β* acts as a protective feedback mechanism that alleviates ER loads and initiates regenerative programs. However, activation of TGF*β* can also amplify *β*‐cell stress eventually resulting in apoptosis and loss of islet mass.^[^ ^102^, ^103^ ^]^ It will be interesting to see whether prolonged hyperglycemia will bias the outcomes of TGF*β* signaling away from regeneration and toward an exacerbation of islet stress.

In the liver compartment of our chips, hyperglycemia induced AKT phosphorylation and resulted in rapid induction of de novo lipogenesis, as well as inhibition of gluconeogenesis, characteristic of the physiological liver insulin response. As no extrinsic insulin was added, these results thus demonstrate functional on‐chip interaction between the two human tissue models. Notably, while SREBP activation was evident in both, liver and pancreas compartments, the majority of differentially expressed genes either did not overlap or were in significant anticorrelation. These results are important as they highlight the tissue‐specificity of metabolic responses and demonstrate the importance of tightly controlling nutrient and hormone levels to understand the crosstalk between regulatory networks. In the liver, we identified hallmarks of increased BCAA catabolism, which has been associated with improved liver function and reduced insulin resistance in chronic liver disease patients.^[^ ^104^ ^]^ By contrast, opposite trends were observed in islets where BCAA stimulate insulin secretion,^[^ ^105^ ^]^ thus perpetuating *β*‐cell stress. Indeed, whether BCAAs are beneficial or deleterious for the maintenance of glucose homeostasis remained controversial.^[^ ^106^ ^]^ Our data suggest that differences in effects are at least in part due to the tissue‐specificity in BCAA catabolic control, emphasizing the added value of analyzing the response of different tissues in an integrated circuit.

Due to its modular architecture, the presented platform can be extended by additional human tissue models thereby being able to further approximate human in vivo metabolic crosstalk in an accessible dynamic system. Particularly, the addition of organotypic models for adipose tissue,^[^ ^107^ ^]^ brain,^[^ ^108^ ^]^ and skeletal muscle,^[^ ^109^ ^]^ which constitute the main glucose sinks besides the liver, promises to result in a model system with intrinsic glycemic control to avoid islet exhaustion. To this end, the established culture conditions offer an ideal springboard as they do not include supplementation with, e.g., growth factors or specific mitogens, which might restrict further tissue compatibility. Furthermore, by using material from different donors, for instance, with and without T2DM, NAFLD or metabolic syndrome, the established model provides an appealing system to parse the molecular basis for interindividual differences in insulin sensitivity and secretion across the glucometabolic spectrum, which are known to be extensive.^[^ ^110^ ^]^

Combined, we have developed a versatile modular chip platform with pneumatic actuation that allows for controlled reciprocal mass transfer between different tissue models at physiologically relevant compartment‐specific flow rates. By establishing an integrated coculture of healthy organotypic primary human liver spheroids and intact primary human islets, we demonstrate functional tissue crosstalk. Importantly, prediabetic hyperglycemia rapidly induced tissue‐specific programs, hallmarked by AKT phosphorylation, de novo lipogenesis, and an inhibition of gluconeogenesis in liver, as well as activation of insulin secretion, UPR, and ER stress in pancreatic islets. Integration of transcriptomic analyses with global transcription factor activity profiling furthermore identified distinct but interacting regulatory networks linking metabolic alterations to signatures of islet regeneration.

## Conflict of Interest

V.M.L. is CEO and shareholder of HepaPredict AB, as well as cofounder and shareholder of PersoMedix AB. R.Z.S., W.v.d.W., and V.M.L. have applied for a patent covering the pneumatically actuated device (GB2594331.A). The other authors do not disclose competing interests.

## Author Contributions

R.Z.S., S.Y. and J.K. contributed equally as joint first authors. W.v.d.W., and V.M.L. contributed equally to this work as joint last author. R.Z.S., W.v.d.W., and V.M.L. designed the microfluidic chip. R.Z.S. and J.K. fabricated the devices. R.Z.S., S.Y., J.K., and J.S. conducted the coculture experiments and analyzed the data. N.T. conducted COMSOL modeling. K.K. performed immunohistochemistry. F.B. analyzed RNA‐Sequencing data. L.C.P. performed mass spectrometry experiments. M.B. fabricated the utilized molds. W.v.d.W. and V.M.L. designed and supervised the study. All authors contributed to the writing of the manuscript.

## Supporting information

Supporting Information

Supplemental Table 2

Supplemental Table 3

Supplemental Table 4

Supplemental Table 5

Supplemental Video 1

Supplemental Video 2

## Data Availability

The data that support the findings of this study are available from the corresponding author upon reasonable request.
