# Supplementary material for: Bioengineered Pancreas–Liver Crosstalk in a Microfluidic Coculture Chip Identifies Human Metabolic Response Signatures in Prediabetic Hyperglycemia
Source: Adv Sci (Weinh). 2022 Oct 26;9(34):2203368. doi: 10.1002/advs.202203368 (PMC9731722; doi:10.1002/advs.202203368)
Supplement: Supplementary file 1 — Supporting Information [file ADVS-9-2203368-s006.pdf]

Supporting Information

for *Adv. Sci.*, DOI 10.1002/advs.202203368

Bioengineered Pancreas–Liver Crosstalk in a Microfluidic Coculture Chip Identifies Human Metabolic Response Signatures in Prediabetic Hyperglycemia

*Reza Zandi Shafagh, Sonia Youhanna, Jibbe Keulen, Joanne X. Shen, Nayere Taebnia, Lena C. Preiss, Kathrin Klein, Florian A. Büttner, Mikael Bergqvist, Wouter van der Wijngaart and Volker M. Lauschke**

Bioengineered pancreas-liver crosstalk in a microfluidic co-culture chip identifies human metabolic response signatures in prediabetic hyperglycemia

Supplementary Text

1. Estimating the average flow rate of the central chamber in an open system (with oil-lock)

The following calculations refer to the chip designs depicted in Figure 1 and Supplementary Figure 1A. Generally, when a syringe pump is the driving mechanism of the microfluidic chip, the commonly known flow rate is the rate of the syringe pump (Q_{syr}). Therefore, we aim to derive the flow rates inside the microchannels based on the syringe pump rate. Although, the hydraulic capacitance of the elastomer tubing is negligible, the compressibility of the actuating air (air in the syringe pump, tubes and the central chamber) causes the instantaneous flow rate of the central chamber to be lower than that of the syringe pump. The narrower and longer the microchannels and/or the higher the syringe pump rate are designed and set, the more significant this flow rate discrepancy is. Nevertheless, it is possible to estimate the average flow rate of the central chamber ($\bar{Q}_c$) throughout a half cycle drive (push or pull).

Naming the pressure and volume of the actuating air as $\mathrm{P}^{act}$and V^{act} respectively, we can overlook the effects of the surface tension and with the assumption of isothermal condition, we describe the ideal gas law between the initial state (time = 0) and the end of the first push cycle (at time = t) for the actuating air as

$$P_0^{act} V_0^{act} = P_t^{act} V_t^{act} \qquad (1)$$

assuming the pressure of the air pockets in the satellite chambers to be maintained close to the atmosphere pressure by the oil-lock system (see Figures 1 and SI.1), we can also define the Hagen–Poiseuille equation for the whole network as

$$\Delta P = Q_c R_m \qquad (2)$$

$$P^{act} - P_{atm} = Q_c R_m \qquad (3)$$

Where ΔP and R_m are the pressure drop and the equivalent hydraulic resistance for the whole network of microchannels respectively. And Q_c is the instantaneous flow rate of the central chamber.

The volume of actuating air at the half cycle time can be defined as

$$V_t^{act} = V_0^{act} - Q_{syr}t + \bar{Q}_c t \qquad (4)$$

In which $Q_{syr}t$ as the volume contracted by the syringe pump during a half cycle (push) and $\bar{Q}_c t$ as the volume of medium pumped out of the central chamber during the same cycle which are subtracted from and added to the initial volume of the actuating air, V_0^{act}.

Owing to the fact that the direction of the medium flow is reversed between the push and pull cycles, Q_c has to be zero at the half cycle time (t) which, in turn, renders the difference between the actuating and atmospheric pressure equal to zero at time t according to Eq.3. Therefore, we can substitute the atmospheric pressure (P_{atm}) for both the initial actuating pressure (P_0^{act}) and the actuating pressure at the half cycle time (P_t^{act}) in Eq.1 and by using Eq.4 we obtain

$$P_{atm}V_0^{act} = P_{atm}(V_0^{act} - Q_{syr}t + \bar{Q}_c t) \qquad (5)$$

$$\bar{Q}_c = Q_{syr} \qquad (6)$$

Which clearly shows that the syringe pump rate is a good approximation for the average volumetric flow rate of the central chamber.

To estimate the flow rates into and out of the satellite chambers, we need to compare the hydraulic resistances of the channels connecting the central chamber to the satellites. In general, the hydraulic resistance for a channel with rectangular cross-section is given by

$$R_h \approx \frac{12\mu L}{d_1 {d_2}^3 (1 - 0.63 d_2/d_1)} : \; d_2 < d_1 \quad (7)$$

Where μ, L are the dynamic *viscosity* of the fluid and the length of the channel, respectively [1]. Also, d_1 and d_2 represent the dimensions of the rectangular cross-section. Thus, the hydraulic resistance of the channel connected to the ith satellite chamber can be estimated as

$$R_i \approx \; \alpha L_i \quad (8)$$

Where L_i is the length of the channel and α ($= \frac{12\mu L}{d_1 {d_2}^3 (1 - 0.63 d_2/d_1)}$) is the same for all the channels due to their equal cross-sectional geometry ($d_1 \; and \; d_2$) and an approximate equal dynamic viscosity of the fluid flowing through them. Therefore, the pressure drop ($\Delta P = P^{act} - P_{atm}$) along each individual microchannel, based on the Hagen-Poiseuille's law, is defined as

$$\Delta P = R_i Q_i = \alpha L_i Q_i \quad (9)$$

Where Q_i is the volumetric flow rate through the ith channel. On the other hand, the same pressure gradient also drives the flow between the central chamber and the whole network of the satellite chambers based on the Eq.2 which results in

$$\Delta P = R_m Q_c = \frac{Q_c}{\sum_{i=1}^{n} R_i^{-1}} \quad (10)$$

In which the equivalent hydraulic resistance of the network (R_m) is defined as the parallel sum of all of the channels' resistances: $R_i s$, (having n number of satellite chambers in general). And by substituting R_i from Eq.8

$$\Delta P = \frac{\alpha Q_c}{\sum_{i=1}^{n} {L_i}^{-1}} \quad (11)$$

Equating Eq.9 and Eq.11, we obtain the volumetric flow rate through each (j^{th}) channel as

$$Q_j = \frac{Q_c}{L_j \sum_{i=1}^{n} L_i^{-1}} \quad (12)$$

Where L_j is the length of the same channel. Flow rates in this equation are instantaneous, however, knowing that the mean flow is the same as the arithmetic averaging of the instantaneous rates, the same expression defines the relation between the average flow rates as well. Thus, combining the Eq.6 and Eq.12 results in

$$\bar{Q}_j = \frac{Q_{syr}}{L_j \sum_{i=1}^{n} L_i^{-1}} \quad (13)$$

This enables us to estimate the mean volumetric flow rate for each individual microchannel, knowing the flow rate of the syringe pump and characteristic lengths of the channels. Similar modeling method holds true for a closed system (without any oil-lock) on condition that the air pockets in satellite chambers are interconnected.

2. Estimating the average flow rate of the central chamber in a closed system (without oil-lock)

For a closed system in which pressure is not maintained at 1 atm, the following calculations are applications are applicable. An exemplary design in which the air pockets of all satellite chambers are interconnected is shown in Supplementary Figure 1B. This leads to a united volume of the air pockets in all the satellite chambers (V^{sat}) with pressure P^{sat} that in turn simplifies the mathematical model. Based on Eq.1 and Eq.4 (main text), the isothermal ideal gas equation for the actuating air can be defined as

$$P_{atm} V_0^{act} = P_t^{act} (V_0^{act} - Q_{syr} t + \bar{Q}_c t) \quad (1)$$

Where P_t^{act} is the pressure of the actuating air at the half cycle time, t. And similarly for the satellite air pockets as

$$P_0^{sat} V_0^{sat} = P_t^{sat} V_t^{sat} \quad (2)$$

$$P_{atm}V_0^{sat} = P_t^{sat}(V_0^{sat} - \overline{Q}_c t) \quad (3)$$

In which P_t^{sat} is the pressure of the satellite air pockets at the half cycle time and V_t^{sat} is defined by subtracting the volume $\overline{Q}_c t$ (injected during the half cycle time to the satellite chambers) from the initial volume of the satellite air pockets. According to Eq.2 (main text), the pressure difference between the actuating and satellite air, at any given time, can be deduced as

$$P^{act} - P^{sat} = Q_c R_m \quad (4)$$

At the half cycle time, t, when the medium flow reverses its direction, the instantaneous flow rate of the central chamber, Q_c is equal to zero, therefore:

$$P_t^{act} = P_t^{sat} \quad (5)$$

Solving the system of linear equations formed by Eq.1, Eq.3 and Eq.5, the average flow rate of the central chamber is

$$\overline{Q}_c = \beta Q_{syr} \quad (6)$$

In which β is the constant of proportionality defined by

$$\beta = \frac{V_0^{sat}}{V_0^{sat} + V_0^{act}} \quad (7)$$

Showing that the average flow rate of the central chamber is proportional to the syringe pump rate and the initial (t = 0) volume ratio of the satellite air pockets to the whole air volume confined in syringe, tubing and the microchip defines the slope of this proportionality.

And finally, to derive the mean flow rates in the microchannels ($\overline{Q}_j$), as discussed previously, the relation in Eq.12 (main text) is, also, valid for the mean flow rates owing to the nature of arithmetic averaging, thus:

$$\overline{Q}_j = \frac{\overline{Q}_c}{L_j \sum_{i=1}^{n} L_i^{-1}} \quad (8)$$

And by substituting $\overline{Q}_c$ from Eq.6:

$$\overline{Q}_j = \left(\frac{\beta}{L_j \sum_{i=1}^{n} L_i^{-1}}\right) Q_{syr} \quad (9)$$

Which means that the average volumetric flow rate in each individual microchannel can be determined knowing the syringe pump rate, the initial air volumes (V_0^{sat} and V_0^{act}) and the lengths of the microchannels.

3. COMSOL Simulation Computational Model

Fluid Dynamics and Chemical Species Transport were modelled in COMSOL (COMSOL, Multiphysics 6.0, Stockholm, Sweden).

3.1 Fluid Dynamics:

To estimate the fluid dynamics through the microfluidic devices, a simplified version of the incompressible Navier-Stokes equation was used:

$$\rho \frac{du}{dt} = -\nabla P + \mu \nabla^2 u \quad (1)$$

$$\nabla \cdot u = 0 \quad (2)$$

where, ρ is fluid density [kg/m^3], u is flow rate [m/s], P is pressure [Pa], and μ is dynamic viscosity [Pa·s]. This modified version of the equation can be used because the Reynold's number is low enough in the device that convective terms become negligible [2]. In this model, the properties of water (at 37°C) were used to estimate those of the media in the experimental setup.

3.2 The Transport of Diluted Solute Interface:

Transport of the chemical species, in this model, was assumed to follow the generic convection-diffusion equation:

$$\frac{dc_i}{dt} + \nabla . J_i + \mathrm{u}. \nabla . c_i = 0 \qquad (3)$$

$$J_i = -D_i \nabla c_i \qquad (4)$$

where c is the solute concentration, D is diffusivity, J is diffusive flux vector and u is the velocity field (solution to the fluid dynamics equations), The model was calculated according to the initial concentrations of the solute from the experiment.

Supplementary Material

Supplementary Tables: 5

Supplementary Figures: 3

Supplementary Videos: 2

Supplementary Text

Supplementary Table 1: Overview of Taqman probes.

**Gene**	**Probe ID**
ALB	Hs00910225_m1
CYP3A4	Hs00604506_m1
CYP2C8	Hs02383390_s1
HNF4A	Hs00604431_m1
G6PC	Hs02560787_s1
PCK1	Hs00159918_m1
GAPDH	Hs02758991_g1

Supplementary Table 2: Differentially expressed genes of on-chip co-cultured pancreatic islets between high glucose and low glucose conditions. Values show FPKM. HG = high glucose (11 mM); LG = low glucose (3.5 mM).

Supplementary Table 3: Activity scores for 500 transcription factors in pancreatic islets on-chip co-cultured in high glucose and low glucose conditions. HG = high glucose (11 mM); LG = low glucose (3.5 mM); SD = standard deviation; TF = transcription factor.

Supplementary Table 4: Differentially expressed genes of on-chip co-cultured liver spheroids between high glucose and low glucose conditions. Values show FPKM. HG = high glucose (11 mM); LG = low glucose (3.5 mM).

Supplementary Table 5: Activity scores for 500 transcription factors in liver spheroids on-chip co-cultured in high glucose and low glucose conditions. HG = high glucose (11 mM); LG = low glucose (3.5 mM); SD = standard deviation; TF = transcription factor.

Supplementary Figure 1

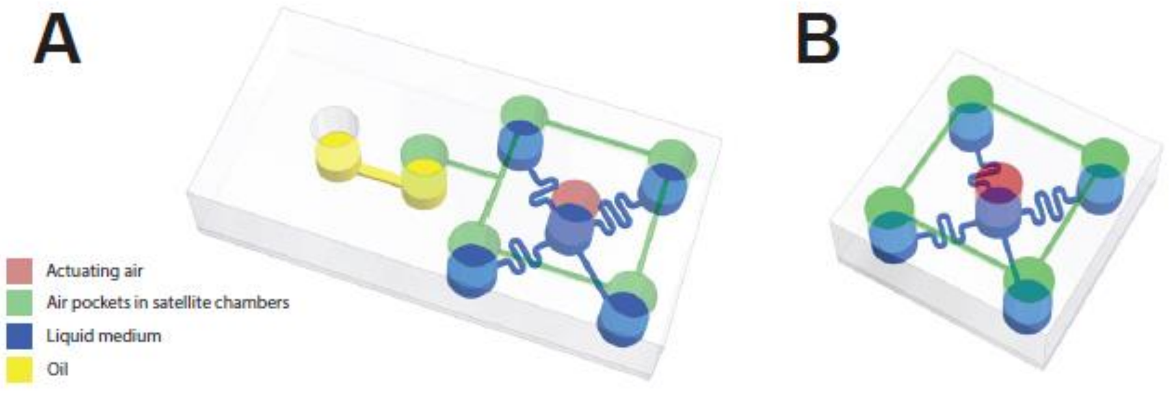

Supplementary Figure 1: Schematic of open and closed microfluidic chip designs. In both incarnations, the chips are comprised of one central and four satellite chambers with air pockets of all satellite chambers being interconnected. **A**, The pressure within the device is maintained at 1 atm by connecting the microchannels to a single oil-lock.

B, Without oil-lock (closed system), the pressure within the device increases over pumping cycles as demonstrated in the Supplementary Text.

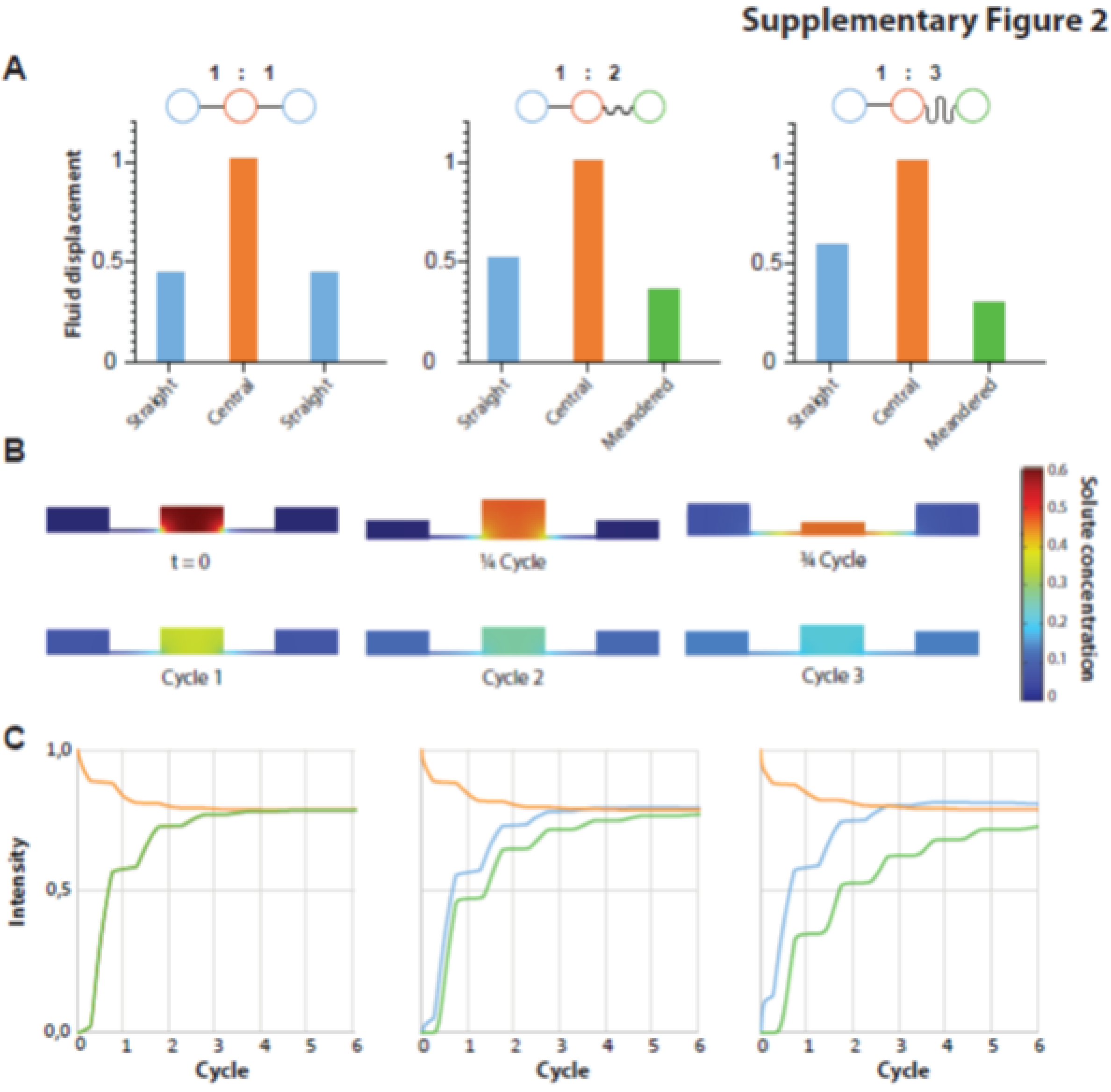

Supplementary Figure 2: Overview of COMSOL modeling results. A, Numerical simulation of fluid displacement for the different compartments with channel ratios of 1:1, 1:2, and 1:3. **B**, Cross-sectional view of the distribution of solute in the chip setup during

different pumping cycles **C**, Computational data representing mixing kinetics and the solute concentration in different compartments during different pumping cycles. Note that mixing kinetics are similar to the experimentally observed profiles.

Supplementary Figure 3

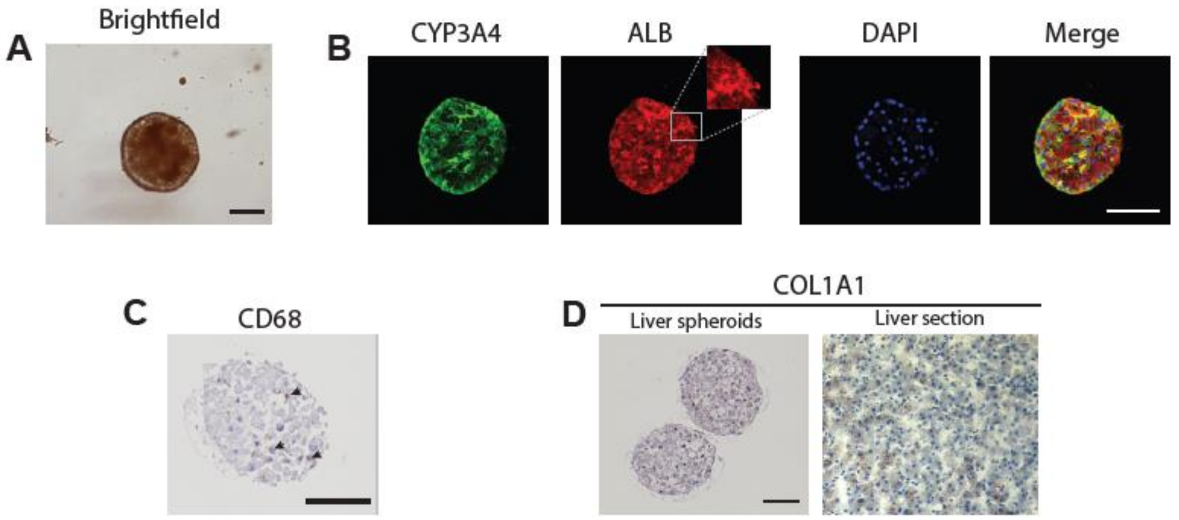

Supplementary Figure 3: Morphological and histological evaluation of liver spheroids. **A**, Representative brightfield image of a human liver spheroid. **B**, Immunofluorescence staining of a liver spheroid for the hepatic markers CYP3A4 and ALB. Note the vesicular staining pattern of albumin. **C**, Immunohistochemistry staining for CD68 indicates the presence of a low number of Kupffer cells (arrow heads). **D**, Staining of liver spheroids shows that COL1A1 localization is diffuse without fibrotic structures, similar to and liver sections from non-fibrotic individuals. Scale bar = 100 µm.

Supplementary Video 1: Numerical simulation of solute transport over time from the central to the peripheral chambers.

Supplementary Video 2: Numerical simulation of solute transport over time from a peripheral compartment to both other compartments.

References

[1] D. E. Angelescu, Highly Integrated Microfluidics Design, 1st Edition, Artech House, Norwood, 2011, p. 159.

[2] S. H. Jung, Y. K. Hahn, S. Oh, S. Kwon, E. Um, S. Choi, J. H. Kang, *Small* **2018**, *14*, e1801731.
